# Clinical profile of 93 cases of 46, XY disorders of sexual development in a referral center

**DOI:** 10.1590/S1677-5538.IBJU.2014.0544

**Published:** 2015

**Authors:** Bianca Costa Mota, Luciana Mattos Barros Oliveira, Renata Lago, Paula Brito, Ana Karina Canguçú-Campinho, Ubirajara Barroso, Maria Betânia Pereira Toralles

**Affiliations:** 1 Universidade Federal da Bahia - Hospital Universitário Professor Edgard Santos, Laboratório de Pesquisa em Infectologia (LAPI), Salvador, BA, Brasil; 2 Universidade Federal da Bahia, Salvador, BA, Brasil; 3 Departamento de Pediatria, Universidade Federal da Bahia, Salvador, BA, Brasil; 4 Departamento de Urologia, Universidade Federal da Bahia, Salvador, BA, Brasil; 5 Departamento de Genética, Universidade Federal da Bahia, Salvador, BA, Brasil

**Keywords:** Epidemiology, Endocrinology, Pediatrics, Cryptorchidism

## Abstract

The term DSD refers to disorders that affect the normal process of sexual development causing disagreement between chromosomal, gonadal and phenotypic sex, and this study aimed to describe the clinical profile of a group with DSD 46, XY joined on DSD Clinic of Hospital of Salvador, Bahia Clinics. It was a retrospective study of medical records of survey data of 93 patients with DSD 46, XY. Among the patients studied 50.5% had no defined etiology and 20.4% had androgen insensitivity syndrome (AIS), 63.4% had been initially recorded in males, 31 (33.3%) in females, being that in two it was necessary to reassignment. All patients with complete AIS pure gonadal dysgenesis and had female genitalia. Others have been diagnosed with genital ambiguity or severe hypospadias and cryptorchidism. The gonads were palpable at the first consultation in 75.3% of patients. It is important to establish an active surveillance program for these patients. The first assessment took place before the age of ten in more than 50% of cases, which shows that much needs to be done for medical education and community about the DSD. Because the phenotypic variability of sexual development disorders was noted that the clinical profile of patients studied ranged between different etiologies, including hindering the diagnostic conclusion of these individuals.

## INTRODUCTION

Individuals with 46, XY karyotype and disagreement between external genitalia and gonadal sex are classified as individuals with 46, XY disorders of sexual development (DSD). Most of these patients have a autosomal recessive pattern of inheritance linked to X chromosome (1, 2).

Patients with 46, XY DSD have lower virilization of genitals compared to normal 46, XY individuals. Etiology may be associated to hypoplasia of Leydig cells, enzyme disturbances of testosterone synthesis, deficit of 5-alfa-reductase enzyme (DEF5α), testicular regression syndrome, gonadal disgenesia (GD), anorquia, androgen insensitivity syndrome (AIS) or ovotesticular DSD (3).

At birth, patients with 46, XY DSD show an array of external genitalia patterns, from a male looking phallus to an almost normal female genitalia with slight increase of clitoris. Testes may be abdominal or at inguinal region. The development of genitals will depend on the capacity of testosterone synthesis of testicles, of the transformation of testosterone in dehydrotestosterone (DHT) by 5-alpha-reductase enzyme or of the presence of receptors sensitive to testosterone. The diagnosis of patients with 46, XY DSD is mainly clinical and laboratorial (4) and the treatment requires a multidisciplinary approach in order to determine the social sex. Besides, these individuals may be referred to certain surgical procedures and hormonal treatment (3, 5, 6).

Initial clinical approach must evaluate the physical characteristics of the individual genitalia. In the presence of ambiguous genitalia (single urethral orifice at the basis of the phallus, non-palpable gonads or presence of gonads at the inguinal region) DSD must be suspected and the urologist must perform a karyotype exam to determine genotypic sex, as well refer the patient to endocrinologists and psychologists (4).

Most published literature regarding DSD is related to patients with XX, DDS rather than XY, DDS. This is a very interesting group of patients, since the presence of chromosome Y and testicles lead to several phenotypes, and adaptation to gender designation (male or female) and or sexual satisfaction may be troublesome. There is a trend that these patients, due to the presence of Y chromosome and probable cerebral masculinization, be characterized as males. However, patients with CAIS (complete androgen insensivity syndrome) and pure gonadal disgenesia (GD) present a better adaptation to female sex (2). The objective of the present paper is to describe the epidemiological and clinical profile of patients with DSD 46, XY syndrome followed in a tertiary referral center ambulatory.

## MATERIALS AND METHODS

This is a retrospective study (approved by the ethical committee, protocol 024/2007) that included the review of the charts of all patients with diagnosis of DSD 46, XY followed in the ambulatory of a tertiary center of disorders of sexual development (state of Bahia, Brazil). Data collection was completed from March to September 2013.

Etiologic diagnosis was based upon clinical and laboratory tests: hormonal profiles, cytogenetic analysis, clinical evaluation of genitalia and pathological exam.

That ambulatory attends referred patients with signs of DSD. At present, it attends 341 patients. Epidemiological data included: birth date, age at first consultation, similar cases among relatives, living at the capital or inner region of Bahia State, sex creation, change of civil registration, age at last consultation, reason of referral and loss of ambulatory follow-up. Abandonment of ambulatory follow-up was considered for those that had not returned for consultation for more than five years.

Clinical aspects included: a description of genitalia according to the size of the phallus, number of orifices, localization of gonads, labioscrotal status and established etiological diagnosis; it was used an Investigative Protocol of 46, XY DSD proposed by the institution. Data were distributed and categorized for each patient in an Excel spread sheet. Target population included 110 elected individuals and the descriptive statistical data were calculated (average, median, interquartile interval for age at diagnosis) and two stratified analyses were proposed. Inferential statistics were not calculated (hypothesis test and confidence interval) since this was a study that covered the whole population.

## RESULTS

Among 93 patients with syndromic diagnosis of DSD 46, XY, 47 (50.5%) had no defined etiologic diagnosis; 19 (20.4%) had AIS (androgen insensitivity syndrome) and 10 PAIS (partial androgen insensitivity syndrome), 16 (17.2%) presented DEF5α, 7 (7.5%) GD (4 pure and 3 mix) and 4 (4.3%) had ovotesticular DSD. 52 of all patients (55.9%) lived outside our city.

Among those 93 patients, 59 (63.4%) had been initially registered as males, 31 (333%) females and there were 3 newborns (3.2%) without civil registration. Initial designation of female sex was observed in 9 (100%) patients with CAIS, 4 without diagnosis, 4(100%) in patients with pure GD, 1 (33.3%) with mix GD, 11 (68.7%) with DEF5α, 1 (25%) with ovotesticular DSD and 1 (10%) with PAIS. In two of these patients it was necessary redesignation of sex. One patient without defined diagnosis was initially considered female and after orthophallusplasty and orchipexy at 13 years old had his civil registration altered to male. Another patient with DEF5α, initially registered as a girl, was submitted at 21 years of age to a masculinizing genitoplasty and had also his civil registration altered to male.

Median age of patients at first consultation was 1 year and 10 months (1.8 years), varying from 4 days to 28 years of age, with interquartile intervals of 7.3 years; 56 patients were referred to ambulatory (60.2%) after the first year of age. The diagnosis of patients with pure GD and CAIS was established in a median age of 7 years and 3 months (varying from 6 months to 27 years). Among those with pure GD 3 (75%) attended the first consultation with more than 14 years old and those 9 patients with CAIS, 2 (22.2%) had more than 12 years old. Among other etiologies, median age was 1 year and 4 months (varying from 11 days old to 29 years), among individuals without defined diagnosis 8 (17%) was more than 12 years at first consultation, among those with DEF5α 1 (20%) was more than 15 years old, with PAIS 1 (10%) was 11 years old and all those with mix GD and ovotesticular DSD were less than 10 years old.

Family history of the occurrence of the same type of DSD was positive in 14 patients (15.05%): 5 (35.7%) had CAIS, 1 (7, 1%) had PAIS, 2 (14.3%) DEF5α, 1 (7.1%) mix GD and 5 (35.7%) had no defined diagnosis. Consanguinity was found in 8 patients: 3 (37.5%) with pure GD, 2 (25%) with DEF5α, 1 (12.5%) with PAIS. Two (25%) had no definitive etiology.

In relation to the aspect of genitals, all patients with CAIS and pure GD had female genitalia. All other were diagnosed with ambiguous genitalia or severe hypospadias and criptorquidism. In patients with mix GD, the median of the size of the phallus at diagnosis was 5cm (varying from 2 to 5.5 cm). In 2 patients, this information was not recorded. Single genital orifice was found in 65 patients (69.9%): 5 were considered boys, 12 as girls and 3 had no information. There were 26 patients with two genital orifices (27.9%) (19 considered boys and 7 girls, and 2 had no information).

Gonads were palpated at first consultation in 70 patients (75.3%). Among these, 32 were unilateral at the inguinal region and 6 were bilateral, at the inguinal region and at the scrotum. All others were in other regions, such as only in the scrotum, abdominal region or the information was not available. Table-1 shows the distribution of palpable gonads among groups with DSD 46, XY.

**Table 1:** Palpaple gonads in different groups of patients with 46, XY DSD.

Etiology	Palpaple gonads			Not informed	Total
	Bilateral	Unilater	No	-	
Without diagnosis	26	09	12	-	47
DEF 5α	10	03	03	-	16
CAIS	05	02	02	-	09
PAIS	07	02	01	-	10
GD	-	-	04	-	04
Mix GD	01	01	01	-	03
Ovotesticular DSD	-	03	-	01	04

**GD** = Gonadal disgenesia; **CAIS** = Complete androgen insensitivity syndrome; **PAIS** = Partial androgen insensitivity syndrome; **DEF5**α = Deficiency of 5α reductase; Ovotesticular DSD-ovotesticular disturbance of sexual develpment.

A total of 35 patients (35.5%) were followed-up. Graph-1 presents the frequency of abandonment of patients of the ambulatory according to etiological diagnosis.

## DISCUSSION

Among all our patients with defined diagnosis, 44% presented AIS, and this was the most frequent diagnosis. Abdullah et al. (7) analyzed 45 cases of 46, XY DSD and 48% had AIS, showing that this condition is the commonest among individuals with 46, XY DSD. Laino et al. (8) studied 46 individuals with 46, XY DSD and 29 (63%) had deficiency of synthesis or androgenic action, 15 (51.7%) showed mutation of the gene of androgenic receptor, suggesting that most had AIS. In the present study, 50% had no defined diagnosis. Other authors agree that many patients with 46, XY DSD are not diagnosed (7, 9, 10). Even with the more available molecular tests that aid the diagnosis of these patients, not always they reach a diagnosis, and, besides very expensive, are not easily available in clinical practice (11, 12).

**Figure 1:**
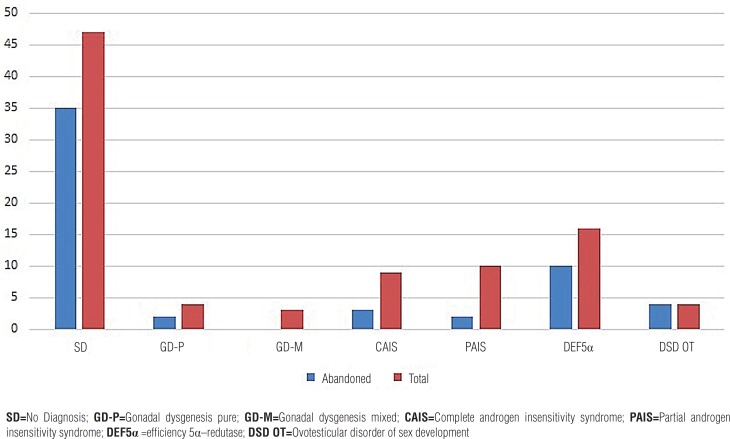
Distribution of patients by diagnosis DsD 46, XY HUPES. **SD**=No Diagnosis; **GD-P**=Gonadal dysgenesis pure; **GD-M**=Gonadal dysgenesis mixed; **CAIS**=Complete androgen insensitivity syndrome; **PAIS**=Partial androgen insensitivity syndrome; **DEF5**α =efficiency 5α–redutase; **DSD OT**=Ovotesticular disorder of sex development

59 patients (63.4%) were registered as males, 31 (33.3%) were raised or registered as females, two had their civil registration altered to male. Andrade et al. (13) described the clinical profile of 62 patients with DSD, and 36 of them were registered as males, and among the 28 patients with X, Y karyotype, only 2 had a female civil registration. Brandão (14) studied 50 patients with DSD 46, XY and 66% were defined as males. Such studies demonstrate that most patients with DSD 46, XY are initially registered as males. All patients with CAIS in our study were registered as females according to their phenotypes and this was confirmed in other researches (10, 15–20).

DEF5α type 2 is a condition with a wide variety of phenotypes, from typical female external genitalia (pseudovaginal hypospadias perineal-scrotal) to a typical male phenotype, without any sign of this condition (11, 21–23). When the patient is considered and designed as female, during puberty it can occur virilization and it may be necessary to redefine the sex as male (24–26). Dessouky et al. study (27) presented 186 cases of DSD 46, XY, and 31 had changed their gender to male and this fact was more frequent among those with DEF5α (52%). Cassia et al. (28) studied 96 cases of DSD 46, XY patients and showed that 20% changed their gender to male. In the present study, among the 16 patients with DEF5α, 11 were registered as females and one of them altered his registration to male. Due to the higher possibility of incongruity of gender definition among individuals with DEF5α, it is more often recommended to designate these individuals as males. Other studies must be carried out in order to confirm that hypothesis in patients with minimal virilization.

Individuals with 46, XY karyotype, disagreement between external genitalia, gonadal and chromosomal sex are classified as carriers of DSD 46, XY syndrome. In those cases, it is necessary an early recognition of the disease, referral to specialists, laboratory tests and surgeries to optimize long term results (2). Treatment of DSD 46, XY individuals requires a multidisciplinary approach and they must be followed along with their families to verify their satisfaction with the gender they are been raised (5, 6). However, although we could count on professionals such as geneticists, urologists, endocrinologists and psychologists, the collected data demonstrated the difficulty to stablish a diagnosis and to follow-up those patients. Although many of them are born with genital abnormalities, only 42% are referred to consultation with few months of life. Abdullah et al. (11) showed that more than 60% of patients with DSD 46, XY are referred to clinical consultation up to one year of age. On the other hand, patients with GD were those who attended the ambulatory more lately in life. Gomes (29) studied 41 female patients with GD 46, XY and all searched medical attention in the second and third decades of life, with primary amenorrhea. Among patients with CAIS, 7 attended firstly the ambulatory with 0 to 9 years old, and 2 at 12 years of age. Late referral usually refers to patients with female phenotypic sex with symptoms of primary amenorrhea at puberty (30, 31). The most precocious diagnosis of CAIS usually occurs due to identification of a testicle during hernia repair surgery in a patient with a phenotypic female genitalia or through palpation of testicles at inguinal region. On the other hand, patients with pure GD are usually diagnosed due to primary amenorrhea or inguinal hernia repair surgery. In our series, several patients with ambiguous genitalia looked for specialized consultation only at puberty. Precocious diagnosis, right after birth, is fundamental, in order to provide medical and psychological counseling to the patient and relatives, to reduce suffering.

Familial history of similar cases were observed in 15.1% of our patients, more frequent in AIS cases (6 patients-42.8%) (1 PAIS and 5 CAIS). Dessouky (27) revised 317 charts of patients with DSD and showed that 210 had DSD 46, XY and among these 36 (17, 1%) had familial history, and higher incidence among patients with PAIS (47.2%) and DEF5α (27.7%). In a study of 33 cases of androgenic insensitivity syndrome, familiar history was positive in 70% of cases (17), this high prevalence of familiar history among patients with AIS may be due to the small number of studied patients in the casuistic and the single nature of the etiology. We suggest that this frequency may vary according to different etiologies but is higher in individuals with AIS.

In the present work, 10% of parents presented consanguinity. The incidence of consanguinity seem higher in countries that allow for endogamy, also, consanguinity is more prevalent in individuals with DSD 46, XY (7, 32). We could not find any paper that described consanguinity rate among patients with 46, XY DSD.

The limitations of the present study include: the retrospective characteristic and the high rate of abandonment of follow-up, and this may imply that the data here presented is related to a selected group of patients.

## CONCLUSION

The disturbances of sexual development 46 XY are rare, and the most frequent etiology in the present study was AIS. The high rate of abandonment of ambulatory follow-up indicates the need for an active search of these patients. First evaluation occurred prior to 10 years of age in more than 50% of cases that warrants for education of medical and lay communities about DSDs. The main reason of referral was genital ambiguity. The clinical profile of patients varied according to etiology, with several phenotypes that made diagnosis difficult. It is recommended to use complementary tools such as molecular biology to evaluate and follow-up patients with 46, XY DSD.
